# A Novel Dynamic Morphed Stimuli Set to Assess Sensitivity to Identity and Emotion Attributes in Faces

**DOI:** 10.3389/fpsyg.2019.00757

**Published:** 2019-04-09

**Authors:** Hayley Darke, Simon J. Cropper, Olivia Carter

**Affiliations:** Melbourne School of Psychological Sciences, The University of Melbourne, Melbourne, VIC, Australia

**Keywords:** face recognition, emotion processing, morphing, dynamic, vision

## Abstract

Face-based tasks are used ubiquitously in the study of human perception and cognition. Video-based (dynamic) face stimuli are increasingly utilized by researchers because they have higher ecological validity than static images. However, there are few ready-to-use dynamic stimulus sets currently available to researchers that include *non-emotional* and *non-face* control stimuli. This paper outlines the development of three original dynamic stimulus sets: a set of emotional faces (fear and disgust), a set of non-emotional faces, and a set of car animations. Morphing software was employed to vary the intensity of the expression shown and to vary the similarity between actors. Manipulating these dimensions permits us to create tasks of varying difficulty that can be optimized to detect more subtle differences in face-processing ability. Using these new stimuli, two preliminary experiments were conducted to evaluate different aspects of facial identity recognition, emotion recognition, and non-face object discrimination. Results suggest that these five different tasks successfully avoided floor and ceiling effects in a healthy sample. A second experiment found that dynamic versions of the emotional stimuli were recognized more accurately than static versions, both for labeling, and discrimination paradigms. This indicates that, like previous emotion-only stimuli sets, the use of dynamic stimuli confers an advantage over image-based stimuli. These stimuli therefore provide a useful resource for researchers looking to investigate both emotional and non-emotional face-processing using dynamic stimuli. Moreover, these stimuli vary across crucial dimensions (i.e., face similarity and intensity of emotion) which allows researchers to modify task difficulty as required.

## Morphed Dynamic Faces

Face processing has been the focus of an enormous variety of research spanning a range of disciplines, including both human, and non-human research. Not only is face processing a rapid, involuntary, and highly specialized perceptual process, but it also plays a crucial role in communication and social interaction. Consequently, face-based stimuli sets are used for a huge range of applications in the study of human perception and cognition, such as investigating low-level visual processing (Cauchoix et al., 2014), memory (Faces subtest, WMS-III; Wechsler, 1997), Theory of Mind (van Veluw and Chance, 2014), different aspects of facial identity perception (Fitousi and Wenger, 2013), and the perception of emotional expression (Fusar-Poli et al., 2009). This final distinction between identity and emotion related processing has represented a particularly strong division in the face processing and literature. Indeed, the widely-accepted dual-route model of face processing argues that these two processes are served by different neural routes (Haxby and Gobbini, 2011; Fitousi and Wenger, 2013).

In addition to exploring these perceptual and cognitive processes in healthy populations, specific impairments in the processing of faces have been demonstrated in certain clinical populations such as schizophrenia (Bortolon et al., 2015), bipolar affective disorder (Bozikas et al., 2006), autism (Aoki et al., 2015), prosopagnosia (Humphreys et al., 2007), and dementia (Bediou et al., 2012). Importantly, the selectivity of the deficits in either identity and emotion processing have been found to vary across clinical disorders affecting different brain areas (Calder, 2011).

Traditionally, face processing studies are typically carried out using behavioral or neuroimaging measures that involve viewing and making judgements about static images of faces. For example, one commonly used measure of identity recognition is the Benton Facial Recognition Task (Benton et al., 1983), which requires participants to match the identity of a target face to one of several possible options. The most widely-used stimulus set for examining emotion processing is the Ekman faces (Ekman and Friesen, 1976), a set of 60 photographs demonstrating the six main emotions: happiness, sadness, disgust, fear, anger, and surprise. Other standardized static face sets include the Japanese and Caucasian Facial Expressions of Emotion (JACFEE; Biehl et al., 1997), the Montreal Set of Facial Displays of Emotion (MSDEF; Beaupré and Hess, 2005) and the Nim Stim Face Stimulus Set (Tottenham et al., 2009).

However, the use of stereotyped or exemplar faces such as these have been criticized for having limited ecological validity. That is, they show only exaggerated, staged expressions which do not reflect the subtler nuances that we experience in natural social interactions (Davis and Gibson, 2000). Furthermore, it has been argued that tasks using exemplar faces are prone to ceiling effects and may not be sensitive to milder impairments in face-specific processes (Harms et al., 2010). To address this, some researchers have utilized morphing software to create stimuli that show varying intensities of emotion. Stimuli such as these permit the study of threshold differences between clinical populations. For instance, it has been shown that patients with schizophrenia require greater intensity to identify expressions such as disgust and fear (Bediou et al., 2005a; Norton et al., 2009; Chen et al., 2012).

In addition to varying intensity, researchers are increasingly utilizing dynamic (i.e., moving, or video-based) face sets to examine face processing impairments. Again, the ecological validity of static stimuli has been questioned because they are not representative of the moving, changing facial expressions we encounter in face-to-face interactions (Kilts et al., 2003; Atkinson et al., 2004). Discerning the emotional state of an individual in daily life involves detecting and rapidly interpreting temporal changes in facial movements such as a brief smile, or a narrowing of the eyes. It can be argued, therefore, that expressions are *inherently* dynamic, and that static images may be too impoverished to adequately tap into emotion processing mechanisms (Fiorentini and Viviani, 2011). An increasing number of dynamic face sets have become available for emotion research, such as the Perception of Emotion Test (POET; Kilts et al., 2003), the Cohn-Kanade Facial Expression Database (Kanade et al., 2000), the CMU-Pittsburgh AU-Coded Face Expression Image Database (Pantic et al., 2005), and the Amsterdam Dynamic Facial Expression Set (ADFES; van der Schalk et al., 2011). Also see Kaulard et al. (2012) and Krumhuber et al. (2017) for further review of available stimuli. It is important to note, however, that these sets typically do not include dynamic non-emotional faces or non-face control stimuli. Indeed, many studies do not include control stimuli at all when examining emotion-processing ability, even when attempting to draw conclusions about the specificity of emotion-processing deficits in clinical populations (for instance, see Bortolon et al., 2015, for a review of the schizophrenia literature).

## Comparison Between Static and Dynamic Faces to Investigate Emotion Processing

Research in healthy controls suggest that there are a range of differences in the ways that individuals respond to static and dynamic face stimuli. In particular, a number of behavioral studies have reported advantages for recognizing emotion from dynamic faces over traditional static faces (summarized in Table 1. Also see Dobs et al., 2018, for a recent review). Several studies found increased accuracy rates for labeling dynamic faces across all emotion compared to matched static faces (Wehrle et al., 2000; Ambadar et al., 2005; Weyers et al., 2006; Calvo et al., 2016). Similarly, a study using dynamic stimuli that varied in the intensity of expression found that dynamic stimuli were recognized more easily than static (Montagne et al., 2007).

**Table 1:** Behavioral studies comparing emotional processing of dynamic and static face stimuli in healthy controls.

**Study**	**Task type**	**Stimuli**	**Dynamic vs. static**
Harwood et al., 1999	Labeling (6 choice)	Real	Dynamic > static (sadness and anger only)
Wehrle et al., 2000	Labeling (10 choice)	Synthetic	Dynamic > static
Kamachi et al., 2001	Rate intensity (7 choice)	Real	Static > dynamic
Ambadar et al., 2005	Labeling (7 choice)	Real	Dynamic > static
Biele and Grabowska, 2006	Rate intensity (4 choice)	Real	Dynamic > static
Weyers et al., 2006	Labeling (6 choice); Rate intensity (7 choice)	Synthetic	Dynamic > static (both measures)
Yoshikawa and Sato, 2006	Matching to same intensity (sliding scale)	Real	No difference, but rapid changes were perceived as more intense than slow changes.
Montagne et al., 2007	Labeling (6 choice)	Real	Dynamic > static
Bould et al., 2008	Labeling (7 choice)	Real	25-frame video > 9-frame video > 2-frame video
Kätsyri and Sams, 2008	Rate each stimulus according to each emotion (6) on a scale of 1–7.	Synthetic and real	Dynamic > static (synthetic faces only). No difference for real faces.
Cunningham and Wallraven, 2009	Labeling (10 choice)	Real	Dynamic > static (except for happy and thinking faces)
Horstmann and Ansorge, 2009	Visual search: Find the negative face in an array of positive faces (or vice versa)	Synthetic	Dynamic > static (faster search times)
Fujimura and Suzuki, 2010	Labeling (6 choice)	Real	Dynamic > static (anger only)
Fiorentini and Viviani, 2011	Labeling (2 choice)	Real	No difference
Recio et al., 2011	Labeling (3 choice)	Synthetic	Dynamic > static (happiness only)
Gold et al., 2013	Labeling (6 choices)	Real	No difference
Hoffmann et al., 2013	Labeling (6 choices)	Real	Dynamic > static (fear and surprise) Static> dynamic (happiness)
Jiang et al., 2014	Labeling (3 choices)	Synthetic	Static > dynamic
Kaufman and Johnston, 2014	Same-or-different discrimination (static only)	Real	Dynamic cues produced faster “same” responses than static cues
Widen and Russell, 2015	Labeling (open-ended responses)	Real	No difference (In children)
Calvo et al., 2016	Labeling (6 choices)	Real	Dynamic > static

Other studies, however, found no difference between static and dynamic conditions (Fiorentini and Viviani, 2011; Gold et al., 2013; Widen and Russell, 2015) or only found significant effects for certain emotions. For example, Recio et al. (2011) found that dynamic happy expressions were recognized with greater accuracy compared to static faces, but reported no such effect for expressions of anger. In contrast, Harwood et al. (1999) reported a dynamic advantage for sad and angry faces (but not happiness, disgust, fear, or surprise), while Cunningham and Wallraven (2009) found a dynamic advantage for sadness, disgust, “clueless” and confused, but not for happiness or “thinking” expressions. In yet another study, Hoffmann et al. (2013) reported a dynamic advantage for fearful and surprised faces, but a static advantage for happy faces. Similarly, Kamachi et al. (2001) found that participants were better at recognizing static faces of anger and sadness compared to dynamic faces. When comparing the recognition of facial expressions presented centrally vs. peripherally, Fujimura and Suzuki (2010) reported that dynamic angry faces were recognized more accurately than static faces in the periphery only, with no differences for central presentation or other valences of emotion.

It is possible that this inconsistency between studies reflects ceiling or floor effects for individual emotions (e.g., happiness reached ceiling in Harwood et al' study), or that the relatively small study sizes in these studies (often *n* < 20) limited the power to detect consistent differences. It has also been demonstrated that varying task instructions can affect the dynamic advantage. For instance, Jiang et al. (2014) found that static faces were recognized faster and more accurately than dynamic faces when participants were placed under time pressure and instructed to prioritize speed of responding. Finally, the “realness” of the face stimuli used may also impact recognition accuracy. One study found a dynamic advantage for computer-generated (synthetic) faces, but no difference between static and dynamic faces for media of real actors (Kätsyri and Sams, 2008). Although the use of synthetic vs. real faces does not appear to draw a consistent pattern across studies (see Table 1), this finding suggests that real and computer-generated faces are not necessarily interchangeable when comparing the properties of dynamic and static stimuli.

In addition to differences in recognition between dynamic and static stimuli, Yoshikawa and Sato (2006) found increased self-reported “emotional experiences” in response to dynamic faces compared to matched static faces. Similarly, Biele and Grabowska (2006) found that dynamic faces were perceived as more intense than static versions. Using a cueing paradigm, Kaufman and Johnston (2014) reported that dynamic cues had a greater impact than static cues on a same-or-different emotion discrimination task. Finally, Horstmann and Ansorge (2009) found that visual search times were improved when searching for a specific dynamic expression among dynamic distractors, compared to search times using an all-static array, suggesting that dynamic faces confer an advantage when rapidly distinguishing *between* different expressions.

Different responses to static compared to dynamic facial expressions have also been demonstrated through imaging studies. In their seminal PET study, Kilts et al. (2003) revealed significantly different patterns of brain activation for dynamic happy and angry faces compared to static, particularly involving area V5, the superior temporal sulcus, cerebellum, and temperomedial cortical areas. LaBar et al. (2003) found increased fMRI activation in the amygdala and fusiform gyrus for angry and fearful dynamic expressions compared to static equivalents, indicating stronger emotional responses to moving (dynamic) stimuli. Similarly, Sato et al. (2004) found greater activation in the fusiform gyrus, medial temporal gyrus, and inferior occipital gyrus in response to dynamic happy and fearful expressions. More recent fMRI studies, including a meta-analysis by Zinchenko et al. (2018), have similarly reported substantial increases in activation to dynamic faces in brain areas associated with the processing of emotion, biological motion, and social cues (Trautmann et al., 2009; Arsalidou et al., 2011; Kessler et al., 2011; Pitcher et al., 2014). Further evidence for a dissociation between static and dynamic facial expressions has come from studies of clinical populations. At least 2 case studies have been published of brain-injured patients who were unable to identify emotions in static images, but could correctly identify emotions from dynamic faces, or from faces formed of moving point-light displays (Humphreys et al., 1993; Adolphs et al., 2003). Taken together, these studies suggest that dynamic faces more effectively tap into neural processes relevant to emotion processing compared to static face images.

Not only do static and dynamic expressions elicit different patterns of behavioral and neural responses, but they also appear to produce differences in viewers' unconscious *muscular* reactions. Electromyography (EMG) studies allow researchers to examine the movements of participants' facial muscles in response to viewing static and dynamic facial expressions. Sato et al. (2008) found that dynamic happy faces prompted stronger activation of the zygomaticus major muscle (involved in smiling), while dynamic angry faces prompted stronger activation of the corrugator supercilii muscle (involved in frowning) compared to static faces. A related study using discreet video recording found similar patterns of muscular movements in response to dynamic faces, suggesting that moving stimuli are more likely to elicit facial mimicry than static images (Sato and Yoshikawa, 2007). Two studies measuring EMG responses to happy, angry, and neutral faces asked participants to rate the intensity of the emotion shown. In both studies, the dynamic stimuli were rated as more intense, as well as more realistic than static equivalents (Weyers et al., 2006; Rymarczyk et al., 2011). However, while happy faces elicited stronger activation of the zygomatic muscles and reduced activation of the corrugator supercilii, no significant EMG differences were found for dynamic angry faces in either study (Weyers et al., 2006; Rymarczyk et al., 2011). Overall, EMG studies suggest that dynamic facial emotions are more likely to prompt spontaneous facial mimicry than static faces, however this finding appears to be more robust for happy faces than for other emotions.

The studies above provide convincing evidence of an advantage for dynamic stimuli over static in the investigation of emotion, at least under certain conditions. However, is this advantage due to the presence of motion, or due to some other characteristic of the stimulus? An obvious confound when comparing dynamic stimuli to static is that they contain different *quantities* of visual information. That is, while dynamic stimuli are comprised of multiple frames, static comprise only one. Therefore, it is possible that dynamic stimuli simply provide a larger number of visual cues compared to static images, and that this drives the recognition advantages seen in previous studies. To investigate whether differences between static and dynamic stimuli still exist when the quantity of information is controlled for, Ambadar et al. (2005) conducted an emotion-recognition study using subtle (low intensity) facial expressions. Performance was compared across four stimulus conditions: dynamic (3 to 6 frame videos, showing a face changing from a neutral to emotional expression), multi-static (the same 3 or 6 frames, with masking in between to eliminate the perception of motion), first-last (two frame videos showing only the first and final image of each previous video, i.e., one neutral face and one emotional face), and single-static (showing the final frame only, i.e., the emotional face). They found that both accuracy and confidence ratings were significantly higher for the two moving conditions (dynamic and first-last) than the two static conditions (single-static and multi-static). This suggests that the advantage shown for dynamic stimuli is due to the presence of motion, rather than the quantity of information. As the performance for the first-last sequence was equal to the dynamic sequence, the authors argue that emotion recognition is likely tuned to the *perception of change* from a neutral face to an expressive face, and is not necessarily dependent on cues relating to the actual temporal unfolding of an expression (Ambadar et al., 2005). However, a follow-up study by Bould et al. (2008) noted that when removing frames from a face video *disrupted* the temporal unfolding of an expression, accuracy decreased. This was interpreted to mean that although the perception of change appears to be a crucial component, accurate expression recognition is also dependent upon the timing of specific muscle movements that build up to the completed expression (Bould et al., 2008).

## Stimuli Development

To permit the assessment of emotional and non-emotional aspects of face processing using comparable stimuli, three matched dynamic stimuli sets were created which combined video with morphing to vary identity and emotion cues (visit http://go.unimelb.edu.au/e3t6 for dynamic stimuli wmv files). These included: a set of emotional faces varying by emotional intensity (fear and disgust); a set of non-emotional face animations varying by similarity (morphing of same-sex or different-sex face pairs); and a set of rotating car animations. This third dynamic set was created to serve as a non-face control stimulus that is matched to the same task parameters as the face sets. Unlike stimuli that are created by morphing two images (i.e., a neutral face to an expressive face, such as those used by Chen et al., 2012), this frame-by-frame morphing procedure retains the more subtle movements seen as the expression unfolds. A video comparing a two-image morphed expression with a frame-by-frame morphed expression can be viewed online in the Supplemental Materials.

Only two negative emotions—disgust and fear—were included in the emotional face set. These were selected because these dynamic expressions are not easily confused with one another in healthy controls (unlike anger and disgust, or fear and surprise; Jack et al., 2014) and, unlike positive expressions, are more likely to elicit emotion-recognition impairments in clinical populations, such as in schizophrenia (Grave et al., 2017) and bipolar disorder (Van Rheenen and Rossell, 2013).

## Step by step creation of new stimuli

### Preparation of Face Stimuli for Morphing

Raw videos were sourced with permission from the MMI-Facial Expression Database (https://mmifacedb.eu; Pantic et al., 2005; Valstar and Pantic, 2010) and the Facial Expressions and Emotion Database (FEED; Wallhoff et al., 2006). Each AVI video file was then converted to a sequence of frames using VirtualDub (Lee, 2012). From this sequence, 13 frames were selected that showed a progression from a neutral resting face to a “completed” facial movement or expression. Each frame was then edited in Adobe Photoshop CS2 to isolate the face against a black background, stabilize any head movements, and to remove non-face cues such as glasses, hair, and facial hair. All stimuli were converted to greyscale to eliminate the possibility of participants using color-matching as an alternative strategy to discriminate between faces. Each face fit within 200 × 200 pixels.

### Morphing to Vary the Intensity of an Expression

**Figure 1:**
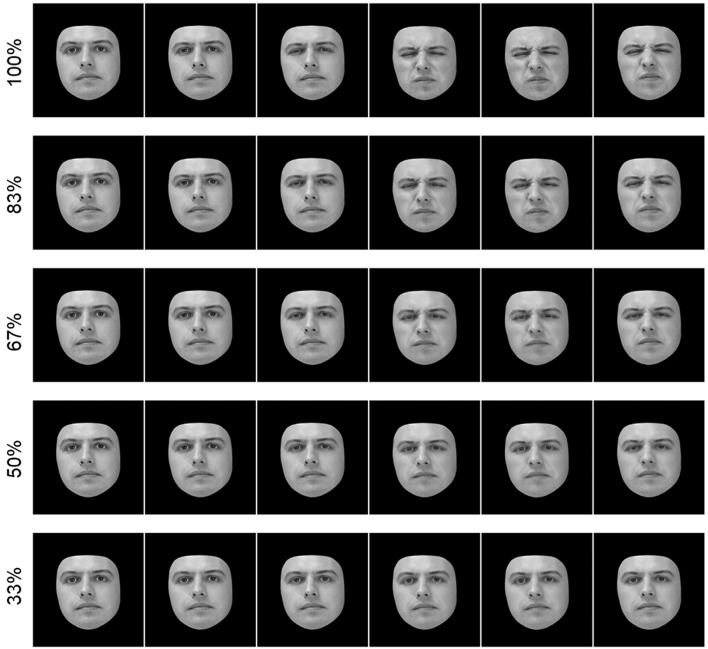
The image above shows partial image sequences (every other frame) from five video stimuli ranging in intensity of emotion. The top video shows the original video of an individual's face changing from neutral to an expression of disgust. This video was then morphed with the neutral face frame (leftmost frame) to reduce the intensity of the final expression (rightmost frame). Emotional intensity ranged from 100% (unedited video) to 33% intensity.

To assess facial affect processing, videos of facial expressions were edited to vary the intensity of each expression without altering the identity of the individual. To achieve this, the first frame of each video (a neutral expression) was morphed with every subsequent frame using Fantamorph 5 (Abrosoft, 2012). This was accomplished using the “Face Locator” add-in to map out the main features of each face. Once the maps were manually adjusted to indicate features as precisely as possible, the morphing slider was used to select the ratio between the neutral and emotive faces, e.g., 33% neutral, 67% emotive. This produced an overall effect of “relaxing” or “diluting” the facial movements in order to create a subtler facial expression in the final video. The final morphed frame was then exported as a new file, and then the process was repeated for all remaining frames. This method was used with videos showing fear and disgust to create a series of animations ranging from 100% intensity to 33% intensity (see Figure 1 for examples). Original animations lasted 1 s. After piloting, however, stimuli were slowed down to two s in order to increase accuracy to an acceptable level.

For this set, 12 videos of different individuals (6 showing fear, 6 showing disgust) were morphed to create five levels of expression intensity: 33, 50, 67, 83, and 100%. A sixth intensity level, 17%, was also piloted. These were not included in the final set, however, because healthy controls could not reliably identify the emotion at such a low intensity. The final set comprised 60 stimuli.

### Morphing to Vary the Identity of a Face

**Figure 2:**
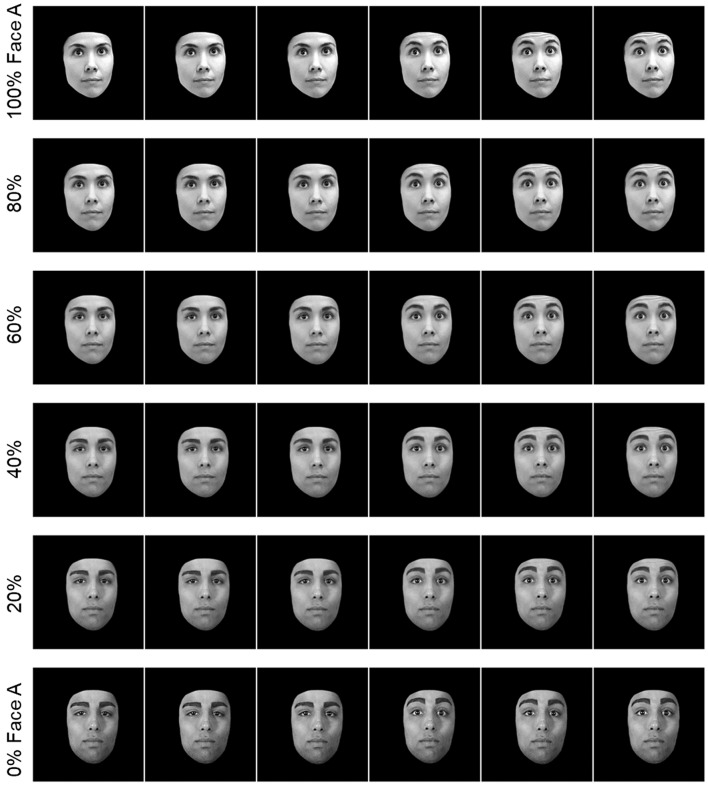
The image above shows partial image sequences from six different videos stimuli. The top and bottom videos show two different individuals making the same motion (eyebrow raise). These videos were then morphed together to create four new videos which vary on a continuum from person A to person B.

To assess *non-emotional* aspects of face processing, videos were edited to vary the similarity between two different individuals. To accomplish this, pairs of videos were selected that showed the same non-emotive facial movement (e.g., raising the eyebrows, opening the mouth, or sticking out the tongue). The thirteen individual frames were then matched as closely as possible so that both videos showed the movement at the same speed. From there, the first frame of one video was morphed with the first frame of the second video using the “Face Locator” add-in in Fantamorph five. When repeated for all frames, this produced a new video showing the new “morphed” individual performing the full facial action. This method was used to create a series of 1 s animations ranging from one individual to the other via 20% increments (see Figure 2 for examples).

Two different sets of stimuli were created: one set where the faces in each morphed pair were the same sex, and one set where each morphed pair were opposite sex. For the same-sex set, 6 pairs of unique individuals (3 male, 3 female) were morphed in pairs to create 6 sets of face animations ranging from one identity to the other by increments of 20%. Thirty-six stimuli were created in total.

The stimuli for the opposite-sex set were created in the same way as above. Six pairs of individuals of the opposite sex were morphed together to create six sets of animations ranging from 100 male to 100% female. Thirty-six stimuli were created. Examples from this set are shown in Figure 2.

### Dynamic Car Stimulus Set

In order to evaluate the specificity of face processing deficits, a set of non-face dynamic stimuli were also created. Side-views of cars were selected because, like faces, they are composed of a fixed configuration of features (e.g., wheels, windows, headlights), but are less likely to invoke face-specific processing networks or tap into emotional responses in the same way as faces. Previous research suggests that, compared to viewing faces, viewing cars evokes significantly less activity in the fusiform face area (Grill-Spector et al., 2004), and do not show the same elevation of the MEG response component M170 (Xu et al., 2005; He et al., 2015).

To create dynamic car videos, 3D meshes of various car models were obtained online via a free 3D modeling website (Studio ArchiDOM, 2011). These meshes were then recolored to match vehicle color and animated using 3DS Max Design (Autodesk Inc., 2012). Each model was animated rotating from a side-view to a 45-degree view. Attempts to use morphing to vary the similarity between cars were unsuccessful. Instead, models were paired with similar looking models in order to avoid ceiling effects in distinguishing different cars.

**Figure 3:**
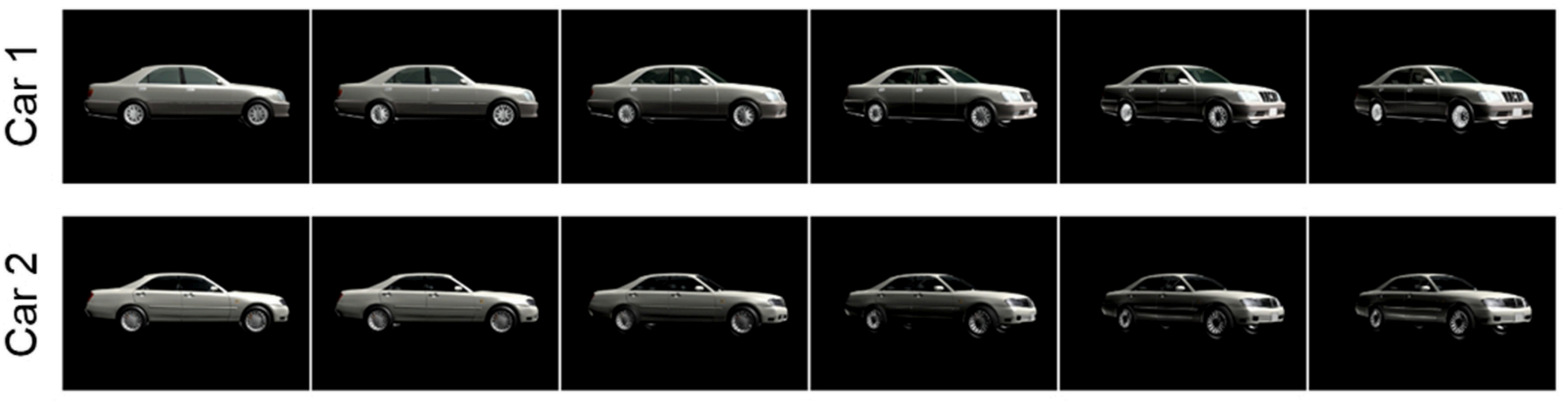
Partial image sequences from two different 3D video stimuli used in the Car Discrimination task. In each video, cars rotate from a side view to a 45-degree view. Car 1 and Car 2 are different models that are similar in appearance.

In total, 6 pairs of 1-s rotating car animations were created. Each of the 12 cars appears in two different animations, once rotating left, and once rotating right. Twenty-four stimuli were created in total. Examples are shown in Figure 3.

## Experiment 1: Verification of Three Dynamic Stimuli Sets Using Different Task Paradigms

Data from twenty healthy controls is presented here to verify these dynamic morphed stimuli using five different tasks: Emotion Discrimination, Emotion Labeling, Identity Discrimination, Sex Labeling, and Car Discrimination. These data were collected as part of a larger study comparing performance across participants with a wide range of psychiatric illnesses (Darke et al., in preparation).

### Method

#### Participants

Twenty healthy individuals (8 male, 12 female) participated in this study. All were free from neurological injury, psychiatric illness and substance use disorder by self-report, and were not taking psychoactive medication. Ages ranged from 18 to 56 (*M* = 34.05, *SD* = 10.72) and all but one was right-handed. Mean formal education was 13.0 years (*SD* = 1.59) and on the basis of performance on the National Adult Reading Test (Crawford et al., 1992), mean estimated WAIS-R FSIQ was 108.7 (*SD* = 6.57). All participants gave written informed consent and received monetary compensation. The study was approved by the University of Melbourne Human Research Ethics Committee (No. 1135478.4).

#### Discrimination Tasks (Same or Different?)

The stimuli sets described above were developed with the intention of creating tasks that are matched on task parameters and vary only in the stimuli presented. One such task is the serially presented same-or-different paradigm. The benefit of this paradigm is that it measures participants' ability to distinguish between stimuli without needing to categorize or label the stimulus shown (Macmillan and Creelman, 1991). That is, participants simply decide if two serially presented stimuli are showing the same emotion, identity, or vehicle, without being required to put a name to the category. To create three versions of the same—or-different task, the morphed emotion stimuli, same-sex identity morphed stimuli, and the car stimuli were presented using the same task parameters. All tasks were programmed using E-Prime 2.0.

##### Emotion discrimination task

Stimuli used were 2-s videos of faces changing from neutral expressions to expressions of either disgust or fear. Original videos consisted of five unique individuals (3 male, 2 female) each showing one expression of disgust and one of fear. These 10 videos were morphed to create five levels of expression intensity (33, 50, 67, 83, and 100%), totaling 50 stimuli.

**Figure 4:**
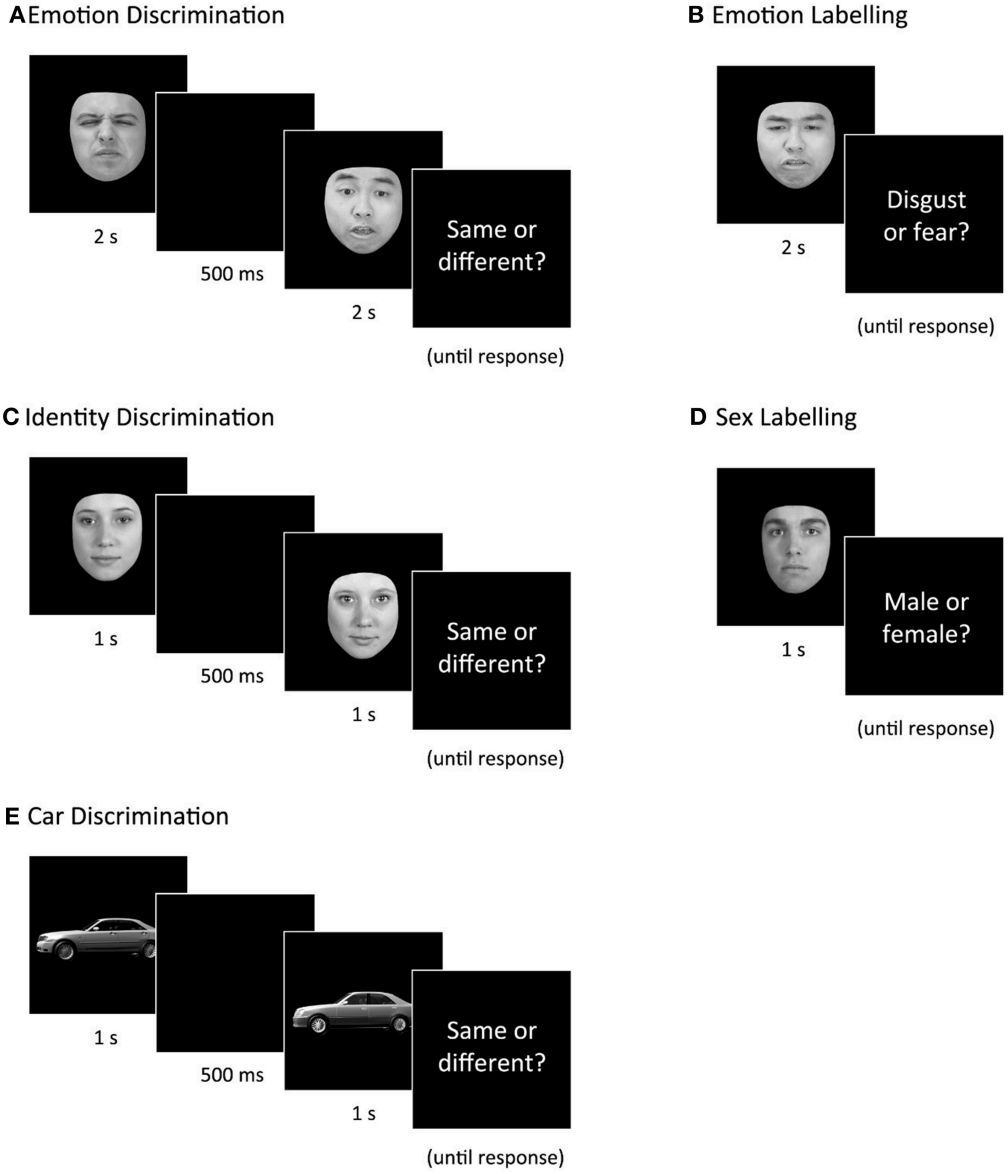
Example trials for each of the five tasks: Emotion Discrimination, Emotion Labeling, Identity Discrimination, Sex Labeling, and Car Discrimination. Correct responses are **(A)** different, **(B)** disgust, **(C)** different, **(D)** male, **(E)** different.

For each trial one expression was shown, then followed by a second face of a different individual showing either the same or different expression, then followed by a blank screen with the words “Same or different?” shown until response (Figure 4A). Pairs of expressions were always shown at the same intensity level. One hundred trials were shown in total.

##### Identity discrimination task

Stimuli used were 1-s videos of faces showing non-emotive facial movements, such as opening the mouth, raising an eyebrow, or poking out the tongue. Stimuli subtended approximately 5.72 × 4.58° of visual angle at a viewing distance of ~50 cm. In order to vary the similarity between pairs of models, video of different individuals (of the same sex) were “morphed” together to create new faces. Six pairs of unique individuals (3 male, 3 female) were used. Each pair was morphed to create six new animations ranging from one identity to the other at 20% increments, totaling 36 stimuli.

For each trial, a “pure” face (either 100% person 1 or 100% person 2) was shown, followed by a second face from the same set that was either 0, 20, 40, 60, 80, or 100% different, then followed by a blank screen with the words “Same or different?” shown until response (see Figure 4C). One hundred and 20 trials were shown in total.

##### Car discrimination task

Stimuli used were 1-s videos of 3D car models rotating from a side view to a 45-degree view. Twelve unique cars were animated and paired with similar looking models. For each trial, one car video was shown, then a second car video was shown, followed by a blank screen with the words “Same or different?” shown until response (Figure 4E). One hundred and twenty trials were shown in total.

#### Labeling Tasks (Fear or Disgust?/Male or Female?)

To determine if varying task demands would produce a different pattern of performance, two different 2-alternative forced choice (2AFC) labeling tasks were also created. These tasks require participants to categorize each stimulus as one of two different categories: male vs. female (for the non-emotion task) or fear vs. disgust (for the emotion task). As recognition tasks do not require comparison of two stimuli (i.e., holding one image in working memory to compare to the next image), they place less load on working memory than same-or-different tasks (Macmillan and Creelman, 1991). This is an important consideration when assessing clinical samples such as schizophrenia, as working memory deficits are increasingly recognized as a central feature of this disorder (Forbes et al., 2009).

##### Emotion labeling task

Stimuli used were the same as those in Emotion Discrimination, with an additional ten videos to make a total of 60 animated stimuli. Each expression was shown for 2 s, then followed by a blank screen with the words “Fear or disgust?” shown until response (Figure 4B). Sixty trials were shown in total.

##### Sex labeling task

Stimuli used were identical to Identity Discrimination above, with the exception that each of the six identities was morphed with an opposite-sex identity instead of a same-sex identity. Six sets of 6 face animations were created, ranging from male to female. Half of the trials were “male” (i.e., 60, 80, or 100% male) and half were “female” (0, 20, and 40% male). For each trial, a single face was shown for 1 s, followed by a blank screen with the words “Male or female?” shown until response (Figure 4D). Each of the 36 faces was shown twice, totaling 72 trials.

#### General Procedure

Participants completed the five tasks in one of four counterbalanced orders. Prior to each task, participants were shown two easy practice trials with feedback. If the participant did not answer the two trials correctly, instructions were repeated and the incorrect trial shown again until the participant understood the task. All responses were given verbally and the examiner logged the response via keypress. In this case, verbal responses were collected in order to: (i) maximize participant engagement during extended testing sessions, and (ii) limit the potential for impulsive responding and accidental key presses (iii) reduce the impact of any known motor impairments in a comparative psychiatric sample (not reported here). Testing took ~90 min to complete, and participants were permitted to take as many breaks as desired. Computerized tasks were completed on a laptop computer (60 Hz, 16-inch screen size) at a comfortable viewing distance of ~50 cm in a quiet, distraction-free environment.

#### Analytical Methods

Participants performing at or below 50% accuracy overall on any task were excluded from further analysis. To evaluate the impact of varying face similarity or emotional intensity, percentage correct was compared across morphing levels within each task. To compare overall performance between tasks, however, *d'* scores were calculated in order to reduce the possible influence of response bias. Percent correct on each task was converted to *d'* scores for each participant using formulae recommended by Macmillan and Creelman (1991). A higher *d'* value indicates more accurate performance. Prior to calculating *d'*, hit rates and false alarms were calculated using formula suggested by Corwin (1994), which are adjusted to avoid dividing by zero. Hit rates were calculated as: (Correct hits + 0.5)/(Total targets + 1); and False alarm rates are calculated as: (False alarm +0.5)/(Total distracters + 1). For the two labeling tasks, *d'* was simply calculated as: *d'* = z(Hit rate)—z(False alarms), where ‘z’ refers to the z transform. The z transforms were calculated using the NORMSINV function in Microsoft Excel. For the three discrimination tasks, this value was then converted to a modified d' using table A5.3 from Macmillan and Creelman (1991). This is because same-or-different tasks are shown to contain an inherent bias to say “same” more often than “different” (Macmillan and Creelman, 1991), therefore a higher level of adjustment is necessary.

A measure of response bias, *c*, can also be calculated using the formula: *c* = −0.5 [z(Hit rate)+z(False alarms)](Macmillan and Creelman, 1991). A value of 0 indicates no bias, while a positive or negative value of c indicates an increasing tendency to favor one response option over the other. D prime and *c* values for each group were then compared using Repeated-Measures ANOVA or mixed ANOVA, where appropriate.

### Results

#### Impact of Morphing (Percentage Correct)

No participants were excluded on the basis of low accuracy. Accuracy across morphing levels was compared using a separate repeated-measures ANOVA for each task.

**Figure 5:**
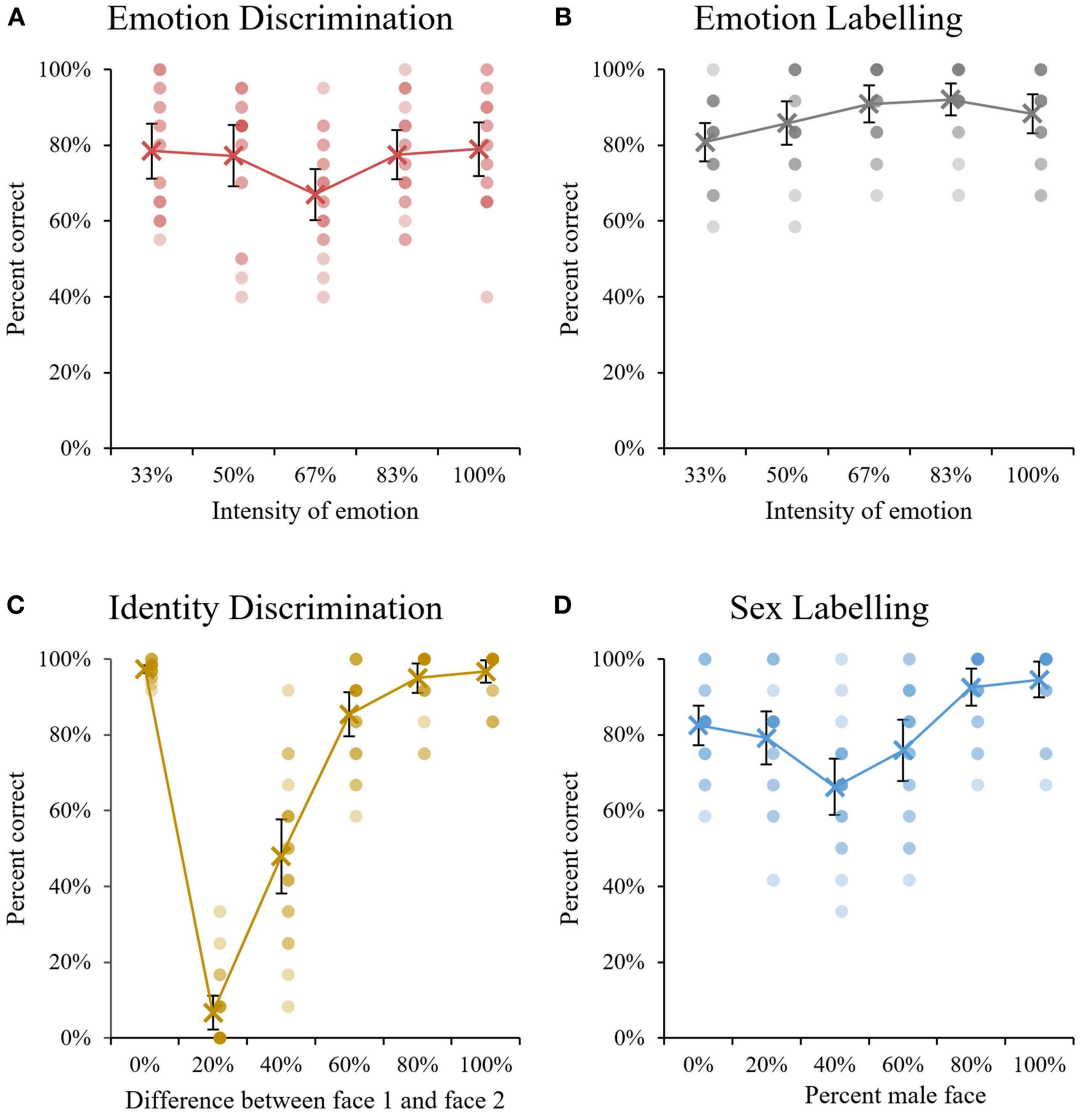
Mean accuracy performance across the five tasks. For **(A,B)**, emotional intensity is presented on the y axis, where 100% indicates an unedited expression and 50% indicates an expression morphed 50% with a neutral expression. For **(C)**, the x axis indicates the degree of similarity between the two faces presented in each same-or-different pair. For **(D)**, the x axis shows the degree of morphing for each condition, where 40% indicates faces that are a 40/60 morph between a male face and a female face. Error bars indicate 95% confidence intervals around the mean and overlapping dots indicate the performance of individual participants.

For Emotion Discrimination (Figure 5A), intensity of emotion had a significant main effect on performance, *F*_(2.40, 45.53)_ = 3.64, *p* = 0.027, $\eta_{\text{p}}^{2}$ = 0.16 (Greenhouse-Geisser corrected). Bonferroni-corrected pairwise comparisons revealed that this was driven by significantly lower performance on the 67% intensity condition compared to all other conditions (*p* = 0.009–0.04). No other comparisons approached significance.

For Emotion Labeling (Figure 5B) the 2 types of emotions were able to be analyzed separately, therefore emotion (fear vs. disgust) was included as an additional factor. The 2 × 5 repeated measures ANOVA showed no main effect of emotion (fear vs. disgust, *F*_(1, 19)_ = 1.07, *p* = 0.32) or interaction between emotion and intensity, *F*_(4, 76)_ = 1.18, *p* = 0.33. However, a significant main effect of intensity was found, *F*_(4, 76)_ = 5.15, *p* = 001, $\eta_{\text{p}}^{2}$ = 0.21. The only pairwise comparison to reach significance showed that performance was significantly lower for 33% intensity faces compared to 83% intensity faces, *p* = 0.009.

For Identity Discrimination (Figure 5C), the degree of similarity between faces had a significant main effect on performance, *F*_(2, 39.45.44)_ = 240.46, *p* < 0.001, $\eta_{\text{p}}^{2}$ = 0.93 (Greenhouse-Geisser corrected). Bonferroni-corrected pairwise comparisons revealed that accuracy for 20% different pairs, 40% different pairs, and 60% different pairs were all significantly lower than the remaining conditions, and significantly different to one another (*p* < 0.001 to *p* = 0.02). Performance on the 0, 80, and 100% different conditions did not differ significantly (*p* > 0.999).

Finally, morphing had a significant main effect on Sex Labeling (Figure 5D), *F*_(1.69, 32.08)_ = 14.83, *p* < 0.001, $\eta_{\text{p}}^{2}$ = 0.44 (Greenhouse-Geisser corrected). Pairwise comparisons showed that accuracy for 40% male faces was significantly lower than all other conditions except 60% male faces (*p* < 0.001). In turn, accuracy for 60% male faces was the next lowest, and significantly lower than 80 and 100% male faces (*p* < 0.001). Finally, accuracy for 20% male faces was significantly lower than 100% males faces (*p* = 0.02). No other comparisons approached significance.

#### D Prime Scores

**Figure 6:**
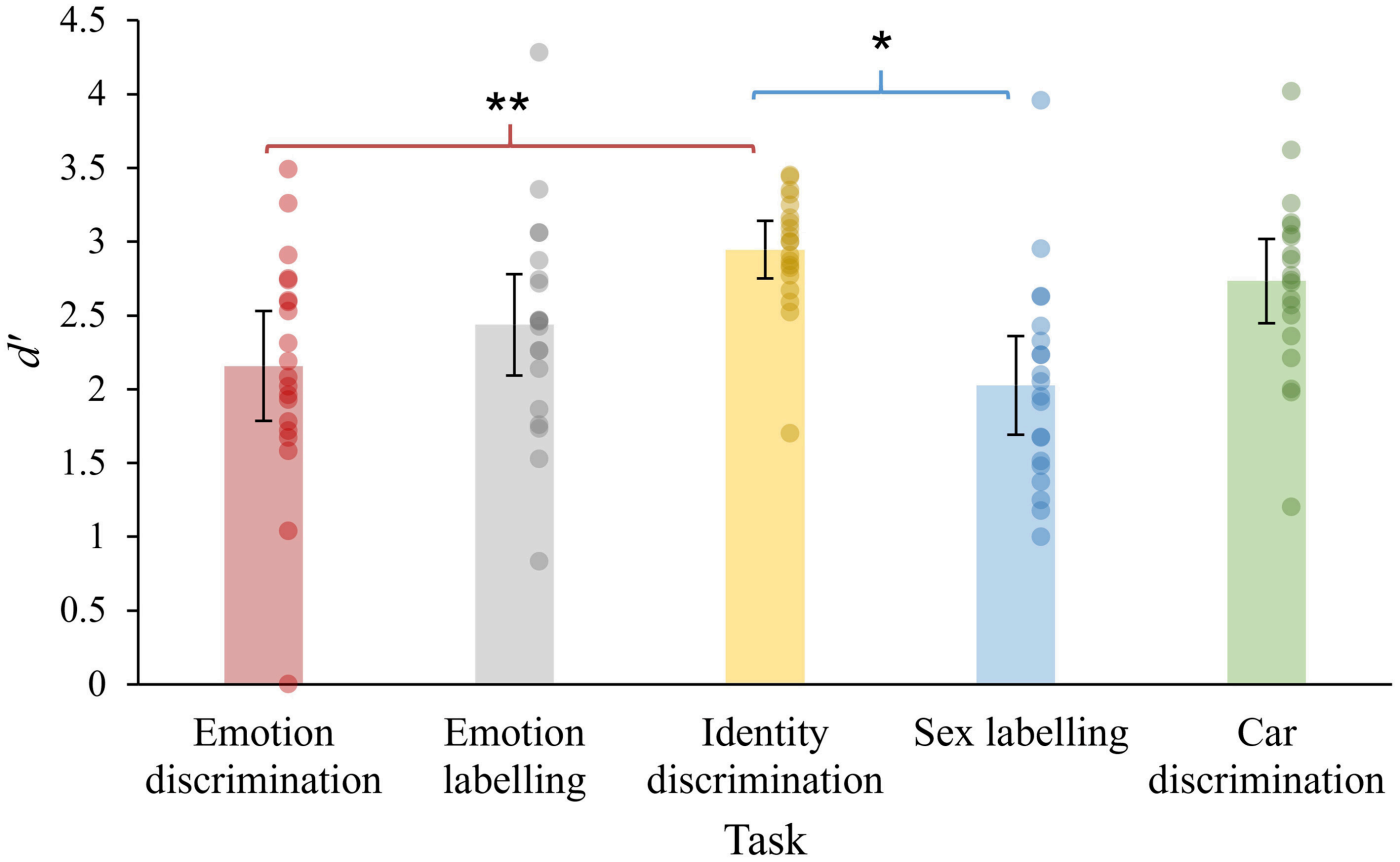
Performance (*d'*) of healthy controls across the five dynamic tasks. Error bars indicate 95% confidence intervals. Dots indicate the performance of individual participants. ^*^p < 0.01, ^**^p < 0.001.

Mean *d'* values ranged from 2.0 to 2.9 across tasks (see Figure 6). To determine whether difficulty varied across the Five dynamic tasks in healthy controls, a repeated-measures ANOVA was conducted with accuracy (*d*') as the dependent variable and task as a within-subjects factor. Mauchly's test showed that the assumption of sphericity was not violated, *X*^2^_(9)_ = 15.64, *p* = 0.08. A significant main effect of task was found, *F*_(4, 76)_ = 7.50, *p* < 0.001, $\eta_{\text{p}}^{2}$ = 0.28, indicating that despite attempts to match task demands, difficulty was not uniform across all tasks. Bonferroni-corrected *post-hoc* tests revealed that performance on Identity discrimination was significantly higher than Sex labeling (*p* < 0.001, *mean difference* = 0.92), and Emotion discrimination (*p* = 0.004, *mean difference* = 0.79). No other comparisons were significant (*p* = 0.07–0.99). This suggests that the Identity recognition task was slightly less difficult compared to the Sex labeling and Emotion discrimination tasks, however performance across all other tasks was of a comparable level.

#### Response Bias (c)

Response bias scores were calculated separately for each task. Response bias scores for the three Discrimination tasks were negative and significantly different from zero, indicating a general tendency for participants to say “same” rather than “different” (Emotion Discrimination: *M* = −0.74, *SD* = 0.11, *t*_(19)_ = 2.48, *p* = 0.02; Identity Discrimination: *M* = −0.74, *SD* = 0.22, *t*_(19)_ = 14.90, *p* < 0.001; Car Discrimination: *M* = −0.67, *SD* = 0.38, *t*_(19)_ = 7.96, *p* < 0.001). For Emotion Labeling, *c* values were not significantly different from zero, (*M* = −0.07, *SD* = 0.32, *t*_(19)_ = 0.35, *p* = 0.35), indicating no bias favoring one response over the other. For Sex Labeling, mean *c*-values were significantly greater than zero (*M* = 0.25, *SD* = 0.40, *t*_(19)_ = 2.81, *p* = 0.01), suggesting a tendency for participants to categorize faces as “male” rather than “female.”

Note that the five included tasks have also been used successfully in a sample of acute psychiatric inpatients with patients able to perform the tasks well-above chance level despite substantial psychiatric symptoms (Darke et al., in preparation). Overall, this preliminary study suggest that the stimuli sets created can be used in a range of paradigms and do not appear to elicit ceiling or floor effects. Interestingly, while morphing appeared to have a linear impact on performance on the non-emotional tasks, no such pattern was found for the emotional tasks. Rather, the effect of varying emotional intensity had a different impact on performance depending on the demands of the task. For Emotion Labeling, intensity had a very limited impact on accuracy, with only one significant difference between conditions (33% intensity emotions recognized less accurately than 83% intensity emotions). Contrary to expectations, this suggests that the morphing technique used to vary intensity had only minimal impact on participants' ability to label disgusted and fearful faces. In contrast, accuracy for the Emotion Discrimination task was comparable across intensity conditions except for an unexpected drop in performance for the 67% intensity condition. This drop in accuracy for medium-intensity faces was unexpected, however it is noteworthy that this dip was no longer evident in the second study using the same stimuli with a larger sample size (*n* = 86, see experiment 2 below).

## Experiment 2: Comparison of Static and Dynamic Stimuli on Two Emotion-Processing Tasks

The aim of this experiment was to determine (a) whether the newly developed set of emotional face stimuli will be identified more easily in dynamic form compared to static form, and (b) whether the type of paradigm used (either labeling or discrimination) will interact with the type of stimulus viewed (dynamic or static) or the level of emotional intensity. To assess this, a group of healthy undergraduate students completed an emotion labeling task and an emotion discrimination task which were each composed of randomly interspersed static and dynamic faces.

### Method

#### Participants

A total of 82 first-year undergraduate psychology students (18 male, 64 female) were recruited from the University of Melbourne. Written informed consent was obtained from all participants, who received course credit in exchange for their participation. According to self-report all participants were free from neurological injury, psychological disorder and substance use, and were not taking psychotropic medications. Ages ranged from 17 to 38 (*M* = 19.60, *SD* = 3.54) and mean formal years of education was 12.23 years (*SD* = 0.76). Due to speaking English as a second language, the estimates of full-scale IQ from the National Adult Reading Test (Crawford et al., 1992) were not available for 28 participants. For the remaining 54 participants, mean estimated WAIS-R full-scale IQ was 112.88 (*SD* = 5.43). Participants also completed the Digit Span Forwards and Backwards subtests from the WAIS-IV (Wechsler, 2008) as a measure of working memory ability. Mean standard score for Digit Span Forwards was 10.02 (*SD* = 2.72) and mean standard score for Backwards was 11.65 (*SD* = 2.92). The study was approved by the University of Melbourne Human Research Ethics Committee (No. 1135478.6). Due to incomplete or missing data, three participants were excluded from analysis for the Discrimination task (*n* = 79) and one was excluded from analyses for the Labeling task (*n* = 81). As all participants performed above 50% accuracy for both tasks, no individuals were excluded on the basis of poor performance.

#### Static vs. Dynamic Emotion Discrimination Task

This task was identical to that described above in Experiment 1, with the exception that half of the stimuli were presented in original dynamic form, and half were changed to static images of the final frame only, presented for 2 s continuously. Stimuli were 5 × 4cm and were viewed at a distance of ~50 cm (5.7 × 4.6° of visual angle). One hundred trials were shown in total.

To attempt to control for the possibility that certain stimuli might produce different effects in the static and dynamic conditions, two different versions were created. In version A half of the faces were presented as dynamic, and half as static. In version B, the stimuli were reversed, with the dynamic stimuli shown as static and the static shown as dynamic. Half of the participants completed version A and half completed version B.

#### Static vs. Dynamic Emotion Labeling Task

This task was the same as described in Experiment 1, except that half of the stimuli were dynamic and half were static images. Participants completed either version A or B to coincide with version A or B of the Discrimination task. That is, for each participant, the faces that appeared as static for the Discrimination task were dynamic for the Labeling task, and vice versa. Sixty trials were given in total.

#### General Procedure

Participants completed the two tasks in one of four counterbalanced orders (either Emotion Labeling or Emotion Discrimination first; either version A or B). Prior to each task, participants were shown two easy practice trials with feedback. If the participant did not answer the two trials correctly, instructions were repeated and the incorrect trial shown again until the participant understood the task. Testing took ~30 min to complete, and participants were permitted to take as many breaks as desired. Computerized tasks were completed on a laptop computer (60 Hz, 16-inch screen size) at a comfortable viewing distance of ~50 cm in a quiet, distraction-free environment.

#### Analysis

Accuracy rates and reaction times in milliseconds were analyzed for each group. D prime scores and *c* values (bias) for each group were compared using *t*-tests and Repeated-Measures ANOVA. Uncorrected reaction times (in ms) for correct trials were also analyzed.

### Results

#### Correlations With Demographics

Uncorrected Pearson correlations were conducted to determine whether performance (*d'*) on either the Discrimination or Labeling tasks were influenced by demographic factors. Correlations were initially run separately for static and dynamic conditions. As coefficients did not differ, data was collapsed across conditions. No significant correlations were found between age, years of education, FSIQ estimates, Digit Span scores, and performance on either of the emotion tasks (*p*s = 0.28–0.99). However, it was found that performance on the two tasks was positively and significantly correlated, *r* = 0.30, *p* = 0.008, CI[.08,0.49].

#### Impact of Task Version

To assess whether the version of tasks completed had any impact on task performance (*d'*) or reaction times, a series of independent *t*-tests were conducted. It was found that participants who completed version B of the tasks were significantly more accurate at labeling static faces than participants who completed version A, *t*_(79)_ = 3.77, *p* < 0.001. No other comparisons approached significance (*p*s = 0.21–0.97). This suggests that the faces shown as static in version A were more difficult to label than the faces shown as static in version B. However, there was no difference in version A or version B faces on the dynamic conditions, or in the Discrimination task for either accuracy or reaction times.

#### Impact of Emotional Intensity

Analyses were conducted to determine whether varying emotional intensity had a differential impact on static and dynamic conditions in either task. A 2 × 2 × 5 Repeated-Measures ANOVA was conducted with percent accuracy as the dependent variable and Task (Discrimination vs. Labeling), Stimuli (Dynamic vs. Static) and Emotion Intensity (33, 50, 67, 83, and 100%) as within-subjects factors. Shapiro-Wilks tests revealed that assumptions of normality were violated (*p* < 0.05) for all 20 conditions. However, upon visual inspection of histograms these were not found to be extreme violations (i.e., data visually approximated the normal distribution and lacked outliers above 2.5 standard deviations; Ghasemi and Zahediasl, 2012). Given equal sample sizes and the robustness of ANOVAs to violations, parametric tests were used for the following analyses. Mauchly's test indicated that the assumption of sphericity was violated for Emotion Intensity $x_{\left(9\right)}^{2}$ = 31.49, *p* < 0.001, therefore the Greenhouse-Geisser correction was used for all analyses involving this factor. Significant main effects were found for Task, *F*_(1, 77)_ = 138.1, *p* < 0.001, $\eta_{\text{p}}^{2}$ = 0.64; Stimuli, *F*_(1, 77)_ = 36.6, *p* < 0.001, $\eta_{\text{p}}^{2}$ = 0.32; and Emotion Intensity, *F*_(4, 261.3)_ = 51.3, *p* < 0.001, $\eta_{\text{p}}^{2}$ = 0.40.

**Figure 7:**
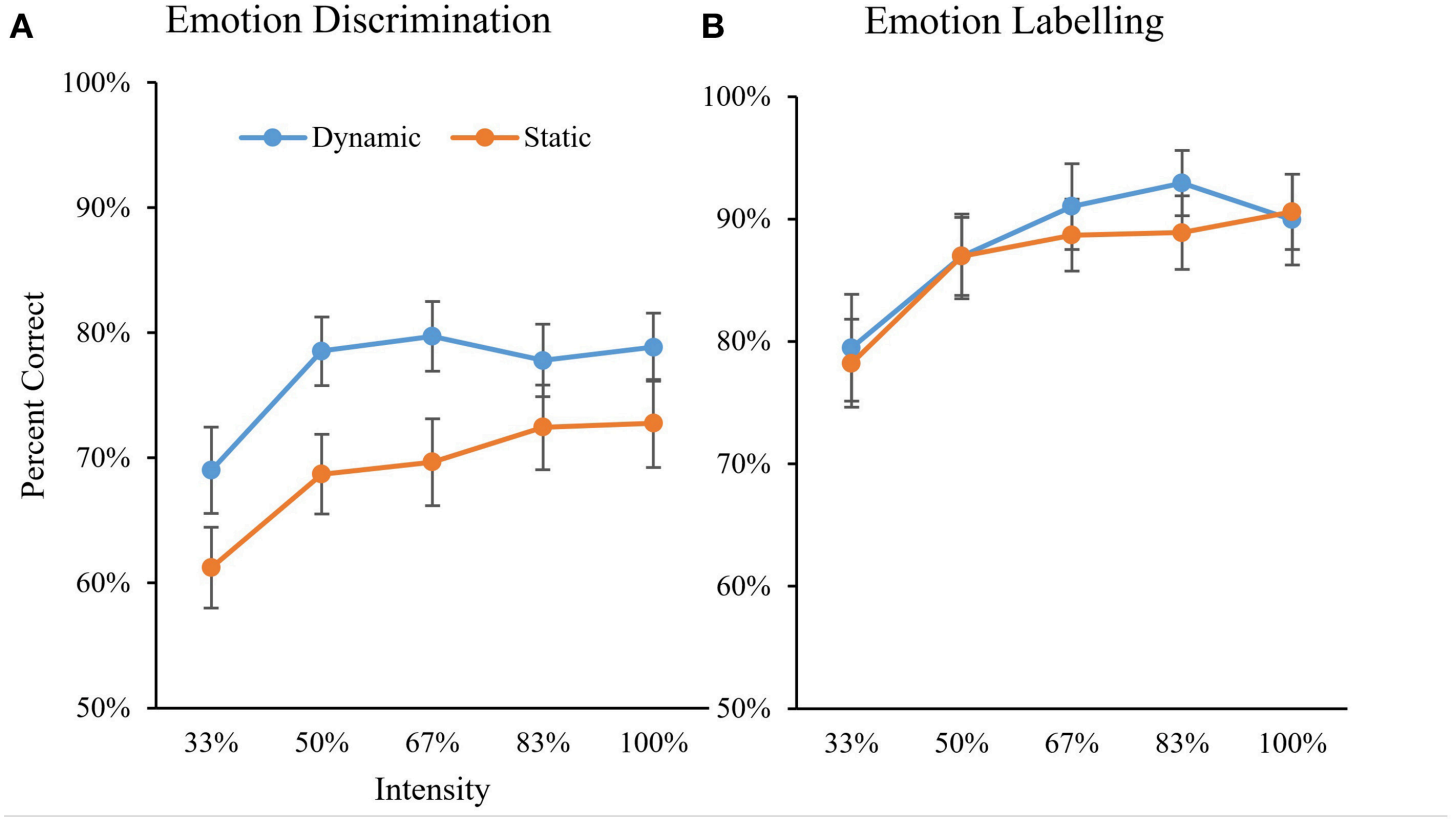
Comparison of percent accuracy by emotional intensity for dynamic and static conditions on the Discrimination task **(A)** and the Labeling task **(B)**. Error bars indicate 95% confidence intervals around means.

Overall, accuracy was higher for the Labeling Task (*M* = 87.4%) than the Discrimination Task (*M* = 72.9%, difference = 14.5%). For both tasks, accuracy increased as Emotional Intensity increased (see Figure 7). Bonferroni-adjusted pairwise comparisons revealed a significant jump in accuracy from the 33% intensity condition to the 50% intensity condition (*p* < 0.001) and a marginally significant jump from the 50% intensity condition to the 67% intensity condition (*p* = 0.056). There was no difference in accuracy between the 67, 83, and 100% intensity conditions (*p*s > 0.99).

A significant interaction between Task and Stimuli was also found, *F*_(1, 77)_ = 11.0, *p* = 0.001, $\eta_{\text{p}}^{2}$ = 0.13. Accuracy was higher for Dynamic stimuli compared to Static stimuli (difference = 7.7%) on the Discrimination Task, *t*_(78)_ = 6.43, *p* < 0.001, but no difference was found for the Labeling Task, *t*_(78)_ = 1.46, *p* = 0.15.

#### Repeated-Measures ANOVA—Accuracy Data (d')

To examine the impact of task and stimulus type on bias-corrected performance rates, a 2 × 2 Repeated Measures ANOVA was conducted with *d'* scores as the dependent variable. Task (Discrimination vs. Labeling) and Stimuli (Dynamic vs. Static) were included as within-subjects factors. As no significant interactions were found involving Emotion Intensity, the data was collapsed across all levels of intensity for the following comparisons. Shapiro-Wilks tests revealed that assumptions of normality were violated (*p* < 0.05) for two of the four conditions, however, once again visual inspection of histograms suggested that these were not extreme violations, and parametric tests were continued.

**Figure 8:**
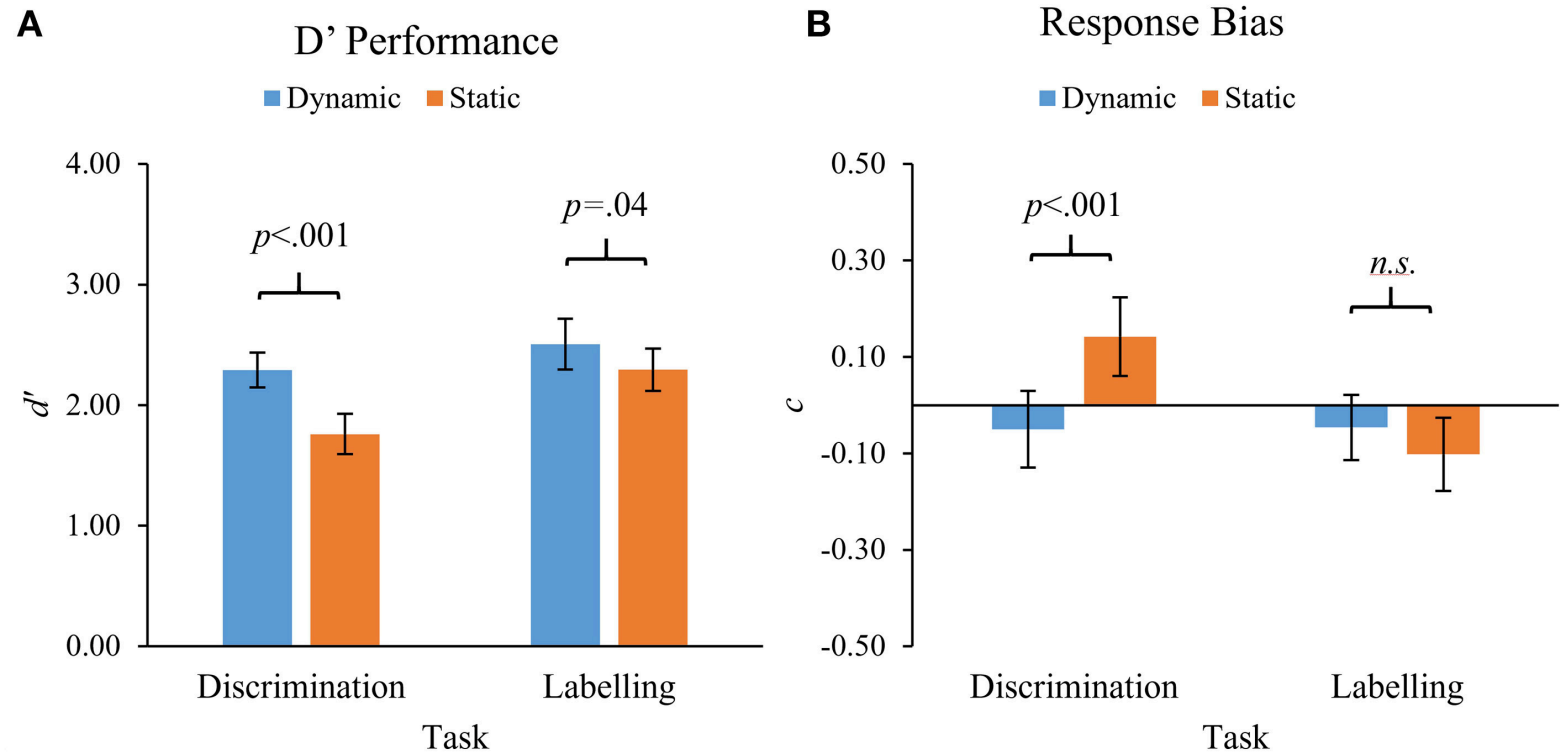
mean *d'* scores **(A)** and mean response bias scores **(B)** for Dynamic and Static stimuli for the 2 tasks: Emotion Discrimination and Emotion Labeling. Error bars indicate 95% confidence intervals.

Significant main effects were found for Task, *F*_(1, 77)_ = 14.5, *p* < 0.001, $\eta_{\text{p}}^{2}$ = 0.16, and Stimuli, *F*_(1, 77)_ = 38.2, *p* < 0.001, $\eta_{\text{p}}^{2}$ = 0.33. Overall, *d'* scores were significantly higher for the Labeling task (*M* = 2.38) compared to the Discrimination task (*M* = 2.02), and were also significantly higher for Dynamic stimuli (*M* = 2.39) compared to Static stimuli (*M* = 2.03; see Figure 8A). A significant interaction between Task and Stimuli was also revealed, *F*_(1, 77)_ = 6.2, *p* = 0.015, $\eta_{\text{p}}^{2}$ = 0.08. This was explored further using *post-hoc* pairwise *t*-tests which showed that, while *d'* scores were significantly higher for Dynamic stimuli compared to Static stimuli on both tasks, this difference was larger for the Discrimination Task (difference = 0.53), *t*_(78)_ = 6.20, *p* < 0.001, than for the Labeling task (difference = 0.21), *t*_(80)_ = 2.14, *p* = 0.04.

#### Response Bias(c)

Mean values of *c* (a measure of response bias) ranged from −0.10 to.14 across the four conditions (see Figure 8B). A 2 × 2 repeated-measures ANOVA with c as the dependent variable and Task (Discrimination vs. Labeling) and Stimuli (Dynamic vs. Static) as within-subject factors was conducted to ascertain whether *c* value differed significantly between the four conditions. A significant main effect was found for Task, *F*_(1, 77)_ = 8.17, *p* = 0.005, $\eta_{\text{p}}^{2}$ = 0.10; but not Stimuli *F*_(1, 77)_ = 3.58, *p* = 0.06, $\eta_{\text{p}}^{2}$ = 0.04. The interaction between Task and Stimuli was also significant, *F*_(1, 77)_ = 12.24, *p* = 0.001, $\eta_{\text{p}}^{2}$ = 0.14. *Post-hoc* pairwise *t*-tests revealed that response bias was significantly greater for Static compared to Dynamic stimuli on the Discrimination task, *t*_(78)_ = −3.87, *p* < 0.001; but no difference was found between Stimuli conditions on the Labeling task, *t*_(80)_ = 1.06, *p* = 0.29. One-sample *t*-tests revealed that *c* was significantly different from zero for Static conditions on the Discrimination task, *t*_(78)_ = 3.42, *p* = 0.001, 95% CI [0.06,0.23], and the Labeling task, *t*_(80)_ = −2.60, *p* = 0.01, 95% CI [−0.18, −0.02]. Mean c values were not significantly different from zero for either of the Dynamic conditions: Discrimination task, *t*_(78)_ = −1.2, *p* = 0.22, 95% CI [−0.13, 0.03], Labeling task, *t*_(80)_ = −1.34, *p* = 0.19, 95% CI [−0.11, 0.02]. This means that for the Discrimination task, participants were more likely to say “different” (rather than “same”) when viewing Static faces, but showed no such bias for Dynamic faces. In contrast, although there was a slight tendency to say “disgust” (rather than “fear”) when viewing Static faces on the Labeling task, this was not significantly different for Dynamic stimuli, which showed no bias.

#### Repeated-Measures ANOVA—Reaction Time Data

**Figure 9:**
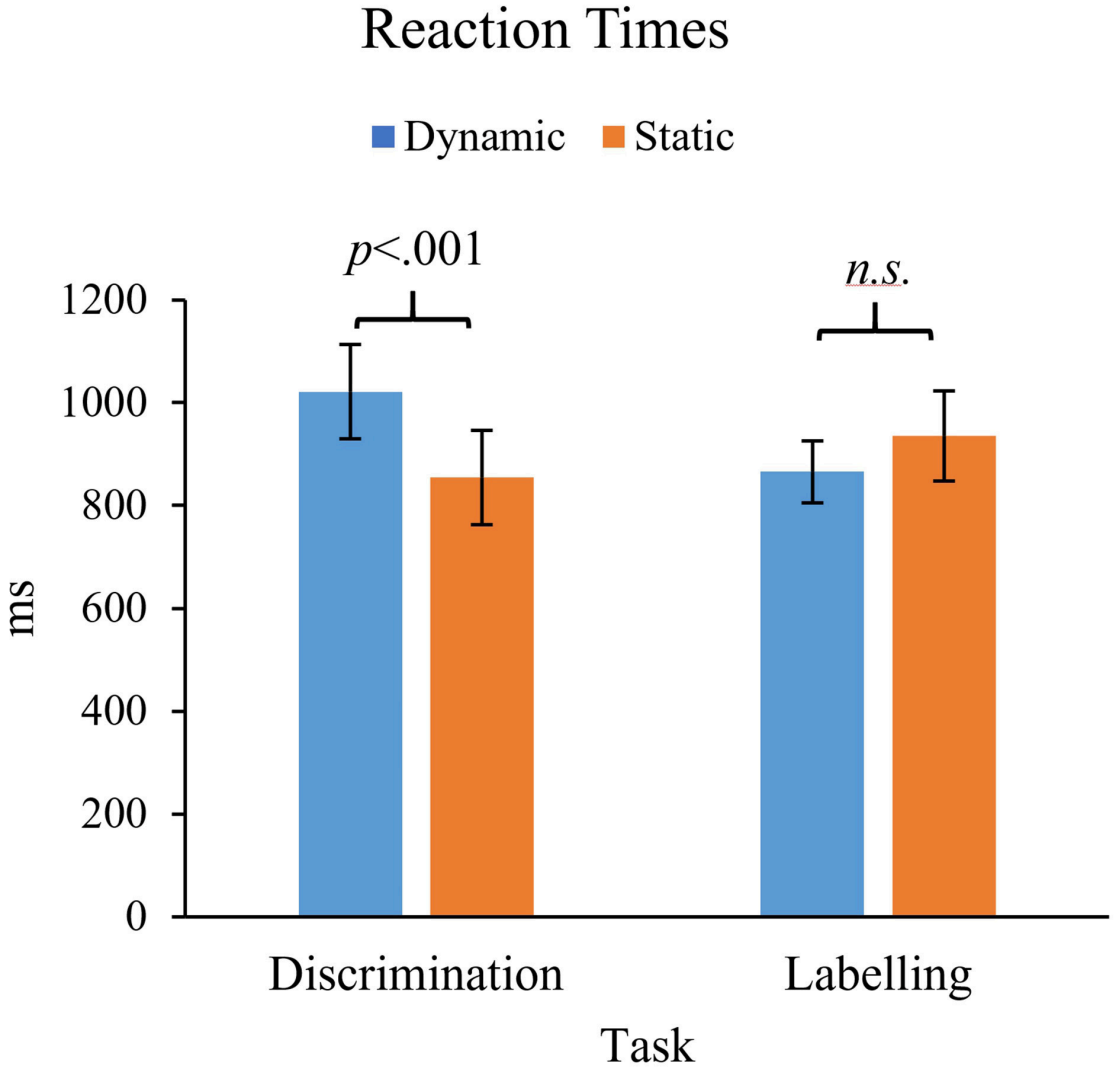
Comparison of reaction times for static and dynamic conditions on the two tasks: Emotion Discrimination and Emotion Labeling.

A 2x2x5 Repeated Measures ANOVA was conducted with reaction times in milliseconds as the dependent variable, and Task (Discrimination vs. Labeling), Stimuli (Dynamic vs. Static) and Emotional Intensity as within-subjects factors. No significant interactions were found involving Emotion Intensity, therefore the data was collapsed across all levels of intensity. Shapiro-Wilks tests revealed that assumptions of normality were violated (*p* < 0.05) for all four conditions, however, once again parametric tests were continued because visual inspection of histograms revealed no extreme violations. Reaction time data are shown in Figure 9. Main effects did not approach significance for either Task, *F*_(1, 77)_ = 0.32, *p* = 0.57, or Stimuli, *F*_(1, 77)_ = 2.34, *p* = 0.13. However, a significant interaction was found between Task and Stimuli, *F*_(1, 77)_ = 9.9, *p* < 0.001, $\eta_{\text{p}}^{2}$ = 0.21. *Post-hoc* pairwise *t*-tests showed that reaction times were significantly slower for Dynamic stimuli compared to Static stimuli on the Discrimination task (difference = 167 ms), *t*_(78)_ = 4.65, *p* < 0.001. In contrast, there was no significant difference between Dynamic and Static conditions on the Labeling task, (difference = 69 ms), *t*_(80)_ = 1.70, *p* = 0.09.

Taken together with the results of the d' analyses, this pattern of results suggests that performance on the Discrimination task was slightly slower, but more accurate for Dynamic stimuli compared to Static stimuli.

## General Discussion

This paper presented three newly-developed stimuli sets which combined dynamic video with morphing in order to assess facial emotion processing, non-emotional face processing, and non-face object processing using comparable stimuli which are sensitive to individual differences in ability. Data obtained in Experiment 1 suggest that these stimuli (emotional, non-emotional, and cars) can be used effectively in both discrimination and labeling paradigms in healthy populations without substantial floor or ceiling effects. Experiment 2 used the emotion set only to compare static and dynamic stimuli using two different task types: discrimination and labeling. Results showed that, as anticipated, performance was higher for dynamic stimuli for both task types, albeit this advantage was less pronounced on the labeling task. Both emotion tasks produced a similar increase in accuracy for stimuli shown at a higher emotional intensity, and this effect was comparable for both dynamic and static stimuli. Interestingly, task performance did not correlate with any demographic factors such as age, years of education, working memory ability, or estimated IQ.

### Dynamic vs. Static Emotions

The finding that dynamic facial expressions were recognized more easily than static equivalents is consistent with a number of past studies comparing these stimuli in healthy populations (Ambadar et al., 2005; Biele and Grabowska, 2006; Montagne et al., 2007; Fujimura and Suzuki, 2010; Recio et al., 2011). This result indicates that participants recognize fear and disgust more readily when it is presented as a moving (dynamic) stimulus rather than a static image. This is in line with the notion that perceived motion is a central component of emotion recognition, and that studies relying on static stimuli alone may be overlooking the importance of this contribution (Alves, 2013). However, an alternative explanation is that the dynamic advantage is not due to the motion itself, but the simple fact that the dynamic stimulus provides more information than a single frame. While this explanation cannot be disregarded, a study by Ambadar et al. (2005) suggests that the dynamic advantage remains even when the quantity of information is controlled for (i.e., by comparing a video to a sequence of static images interspersed with masks to disrupt the perception of motion). Therefore, it does not seem likely that the results seen in the current study are due to factors unrelated to the perception of motion.

### Discrimination vs. Labeling Paradigm

To the best of our knowledge, this is the first study to compare static and dynamic faces on an emotion discrimination task. Interestingly, this task not only showed a dynamic advantage, but this advantage was more pronounced than that shown in the labeling task. It could be argued that this discrepancy is due to an underlying difference in difficulty between the two tasks, as accuracy was higher for the labeling task overall. However, there was no difference in accuracy rates between the dynamic conditions for each task, which suggests that the two tasks were reasonably well-matched in difficulty for the dynamic stimuli. One could argue, instead, that performance on the discrimination task was more affected by a *loss of motion* than performance on the labeling task. In other words, same-or-different discrimination tasks may simply be a better measure of motion-sensitive emotion processing ability. Further research is clearly required to investigate this idea, and is beyond the scope of this paper. Nevertheless, it would be informative to know if this result is replicated in other studies. For instance, the dynamic advantage has not been unanimously found across studies, and it could be due to the paradigm used. In contrast to the experiments cited above, some studies using dynamic labeling paradigms have reported no dynamic advantage (Fiorentini and Viviani, 2011) or only found an advantage for certain emotions, such as anger or happiness (Fujimura and Suzuki, 2010; Recio et al., 2011). It is possible that labeling tasks may simply be less effective at tapping into emotion processing deficits than other paradigms, such as discrimination.

### Impact of Emotional Intensity

Contrary to Experiment 1, which showed almost no impact of varying emotional intensity, Experiment 2 showed that accuracy increased with higher emotional intensity for both the discrimination and labeling tasks. The reason for this discrepancy is unclear. The lack of pattern shown in Experiment 1 may reflect reduced power due to the smaller sample size (*n* = 20). Alternatively, it may be that the presence of both static and dynamic stimuli in the Experiment 2 tasks prompted participants to adopt a different type of strategy that somehow increased the importance of emotional intensity in these tasks. Regardless, in Experiment 2, similar patterns of increasing accuracy with higher intensity were shown for both dynamic and static stimuli, with no evidence of an interaction between intensity and type of stimulus. This finding is consistent with the study by Hargreaves et al. (2016) who reported similar patterns using dynamic expressions in an emotion labeling task. It is also consistent with studies using static stimuli of varying emotional intensity (Bediou et al., 2005b; Norton et al., 2009; Chen et al., 2012). On closer inspection, this increase in accuracy in the current study appears to be driven by jumps between the 33 and 50% intensity conditions, and between the 50 and 67% conditions. Accuracy did not differ between the 67, 83, and 100% conditions for either static or dynamic stimuli. This result is unlikely to be due to ceiling effects, as raw accuracy in these conditions was below 80% for the discrimination task and below 90% for the labeling task. Therefore, it is possible that participants did not obtain more useful visual information from the most intense expressions. Whether this reflects constraints imposed by the limited presentation times, or some other inherent difficulty in distinguishing fearful and disgusted faces in this stimulus set remains unclear. Although one of the goals of this project was to create stimuli that do not elicit ceiling effects, researchers wishing to achieve 100% performance with these stimuli may wish to explore this by extending presentation times in their own paradigms.

## Conclusions and implications

Video-based face stimuli have higher ecological validity than traditional static images, yet there are few dynamic stimulus sets currently available to researchers that include non-emotional faces and non-face control stimuli. Here we present three sets of matched dynamic video stimuli depicting emotional faces (fear and disgust), non-emotional faces, and cars. These stimuli have been created using morphing software to vary stimulus parameters such as the intensity of a facial expression, and the similarity between faces of different individuals. This allows researchers to have greater control over these parameters in order to create tasks that are more sensitive to individual differences in performance. Using these stimuli, our study found that dynamic stimuli were processed more accurately than static faces for two types of emotion-processing tasks, and that this advantage was greater for a discrimination paradigm than the more commonly-used labeling paradigm. This suggests that these new stimuli are an effective tool to assess emotion processing deficits compared to traditional static image sets. These stimuli are available to download from http://go.unimelb.edu.au/e3t6.

## Ethics Statement

This study was carried out in accordance with the recommendations of the Melbourne School of Psychology Sciences Human Ethics Advisory Group (HEAG) with written informed consent from all subjects. All subjects gave written informed consent in accordance with the Declaration of Helsinki. The protocol was approved by the University of Melbourne Human Research Ethics Committee (No. 1135478.6).

## Author Contributions

HD, SC, and OC contributed conception and design of the study. HD performed the statistical analysis and wrote the first draft of the manuscript. All authors contributed to manuscript revision.

### Conflict of Interest Statement

The authors declare that the research was conducted in the absence of any commercial or financial relationships that could be construed as a potential conflict of interest.
